# Joint Modeling of RNAseq and Radiomics Data for Glioma Molecular Characterization and Prediction

**DOI:** 10.3389/fmed.2021.705071

**Published:** 2021-08-19

**Authors:** Zeina A. Shboul, Norou Diawara, Arastoo Vossough, James Y. Chen, Khan M. Iftekharuddin

**Affiliations:** ^1^Vision Lab, Department of Electrical & Computer Engineering, Old Dominion University, Norfolk, VA, United States; ^2^Department of Mathematics & Statistics, Old Dominion University, Norfolk, VA, United States; ^3^Department of Radiology, Children's Hospital of Philadelphia, University of Pennsylvania, Philadelphia, PA, United States; ^4^University of California, San Diego Health System, San Diego, CA, United States

**Keywords:** RNA sequencing, radiomics, radiogenomics, negative binomial, molecular mutation

## Abstract

RNA sequencing (RNAseq) is a recent technology that profiles gene expression by measuring the relative frequency of the RNAseq reads. RNAseq read counts data is increasingly used in oncologic care and while radiology features (radiomics) have also been gaining utility in radiology practice such as disease diagnosis, monitoring, and treatment planning. However, contemporary literature lacks appropriate *RNA-radiomics* (henceforth, ***radiogenomics***) joint modeling where RNAseq distribution is adaptive and also preserves the nature of RNAseq read counts data for glioma grading and prediction. The Negative Binomial (NB) distribution may be useful to model RNAseq read counts data that addresses potential shortcomings. In this study, we propose a novel radiogenomics-NB model for glioma grading and prediction. Our radiogenomics-NB model is developed based on differentially expressed RNAseq and selected radiomics/volumetric features which characterize tumor volume and sub-regions. The NB distribution is fitted to RNAseq counts data, and a log-linear regression model is assumed to link between the estimated NB mean and radiomics. Three radiogenomics-NB molecular mutation models (e.g., *IDH* mutation, *1p/19q codeletion*, and *ATRX* mutation) are investigated. Additionally, we explore gender-specific effects on the radiogenomics-NB models. Finally, we compare the performance of the proposed three mutation prediction radiogenomics-NB models with different well-known methods in the literature: Negative Binomial Linear Discriminant Analysis (NBLDA), differentially expressed RNAseq with Random Forest (RF-genomics), radiomics and differentially expressed RNAseq with Random Forest (RF-radiogenomics), and Voom-based count transformation combined with the nearest shrinkage classifier (VoomNSC). Our analysis shows that the proposed radiogenomics-NB model significantly outperforms (ANOVA test, *p* < 0.05) for prediction of *IDH* and *ATRX* mutations and offers similar performance for prediction of *1p/19q codeletion*, when compared to the competing models in the literature, respectively.

## Introduction

Radiomics is increasingly being applied to radiology practice in disease diagnosis, grading, monitoring, and treatment planning (1, 2). Radiomics is extracted from various radiological images of a targeted area of the disease. Fusing the important radiomics and genomics information in the proper computational machine learning (ML) model may helpto achieve a more comprehensive disease diagnosis, prognosis, and treatment planning scheme (3–5). Different studies have evaluated the association between glioma molecular subtypes and radiomics (e.g., tumor shape and size) (6–8), or between different form of genomics (e.g., RNA sequencing (RNAseq) gene expression, protein expression, copy number, molecular mutations, or DNA methylation) and glioma subtypes (9–11).

Conventional ML models do not adequately model the count-based nature of the RNA-sequence data as these models are usually designed to work with data that has a normal distribution. In order to alleviate the lack of appropriate ML models, researchers propose to transform the RNAseq read-count data to approximate a normal distribution. The transformation to normal distribution allows the use of existing methods such as the nearest shrinkage method (12, 13) or Random Forest for classification. However, such transformation removes the count-based nature of the RNAseq read counts data, and hence, lacks the ability to fully preserve the strong mean-variance relationship that is otherwise useful for glioma classification and prediction (14, 15). In order to appropriately model RNAseq read-count data, Negative Binomial (NB) and Poisson distributions are commonly used (16). The Poisson distribution is a single parameter distribution with its mean equals to its variance, which makes it rather restrictive. On the other hand, NB is similar to a Poisson distribution with an additional parameter called “dispersion” that allows the NB distribution to modify its variance without affecting the mean.

RNAseq uses high-throughput or next-generation sequencing technology (NGS) and has emerged as a novel alternative to microarray-based techniques for quantifying gene expression. The microarray technique is known to suffer from background noise. Gene expression level is measured as the relative frequency of the RNAseq reads that are mapped to one gene (17). RNAseq is a very sensitive technique that provides high resolution and a thorough understanding of the transcriptome and has revealed many novel gene structures.

RNAseq distribution requires an appropriate model that adapts and preserves the nature of RNAseq read counts data, and such classification models that preserve the nature of RNAseq are lacking in the traditional ML literature. The NB distribution is an appropriate choice to model such discrete reads counts data (16). Even though traditional ML tools that are developed based on NB are lacking, the choice of using NB distribution in differential gene expression and RNAseq analysis has been adapted by different studies in the literature such as in EdgeR (18–20), DESeq (21), and NBPSeq (22).

An example of a count-based classifier that fits a NB distribution is the Negative Binomial Linear Discriminant Analysis (NBLDA). NBLDA is a well-known classifier that is developed by fitting NB to RNAseq and the mean and dispersion parameter are estimated from the RNAseq data (23). A different type of classifier, known as VoomNSC, is developed based on the transformed count data. VoomNSC is a combination of Voom (an acronym for mean-variance modeling at the observational level) transformation (12) and the nearest shrunken centroids classifier (NSC) (24).

Consequently, the aim of this work is to implement a joint radiogenomics-NB model that predicts and classifies glioma molecular mutations following the 2016 World Health Organization's (WHO) updated guidelines for classification of tumors of the Central Nervous System (CNS) (including high grade and diffuse low-grade gliomas) (25). This work is critical especially when the RNAseq of some cases are unknown and a careful assessment is needed to avoid mischaracterization of lower grade gliomas. In this work, we utilize both volumetric features (radiomics) and RNAseq to implement and learn a radiogenomics-NB model. Then, the trained radiogenomics model is used to predict and classify the unknown RNAseq data. In the proposed model, a log-linear regression modeling is fitted to the estimated mean of the NB distribution and is linked with radiomics. We introduce this step to fuse the continuous radiomics data with the RNAseq count-based data without the need to transform RNAseq data into a normal distribution. Finally, we compare our radiogenomics-NB model performance with that of different genomics and radiogenomics state-of-the-art methods in the literature.

The rest of the paper is organized as follows. A complete step-by-step mathematical derivation of the radiogenomics-NB model and parameters' estimations are presented in section Methodology. Section Experimental Results addresses the dataset used in this study, the data preparation, and the effect of using different numbers of differentially expressed genes in the radiogenomics-NB model. Furthermore, in section Experimental Results, a comparative analysis is discussed in which we compare the proposed radiogenomics-NB model's performance with different well-known methods in the literature. Moreover, in section Experimental Results, we investigate the effect of gender by developing a gender-specific radiogenomics-NB model for glioma molecular grading. Finally, the study's discussion is addressed in section Discussion.

## Methodology

**Figure 1:**
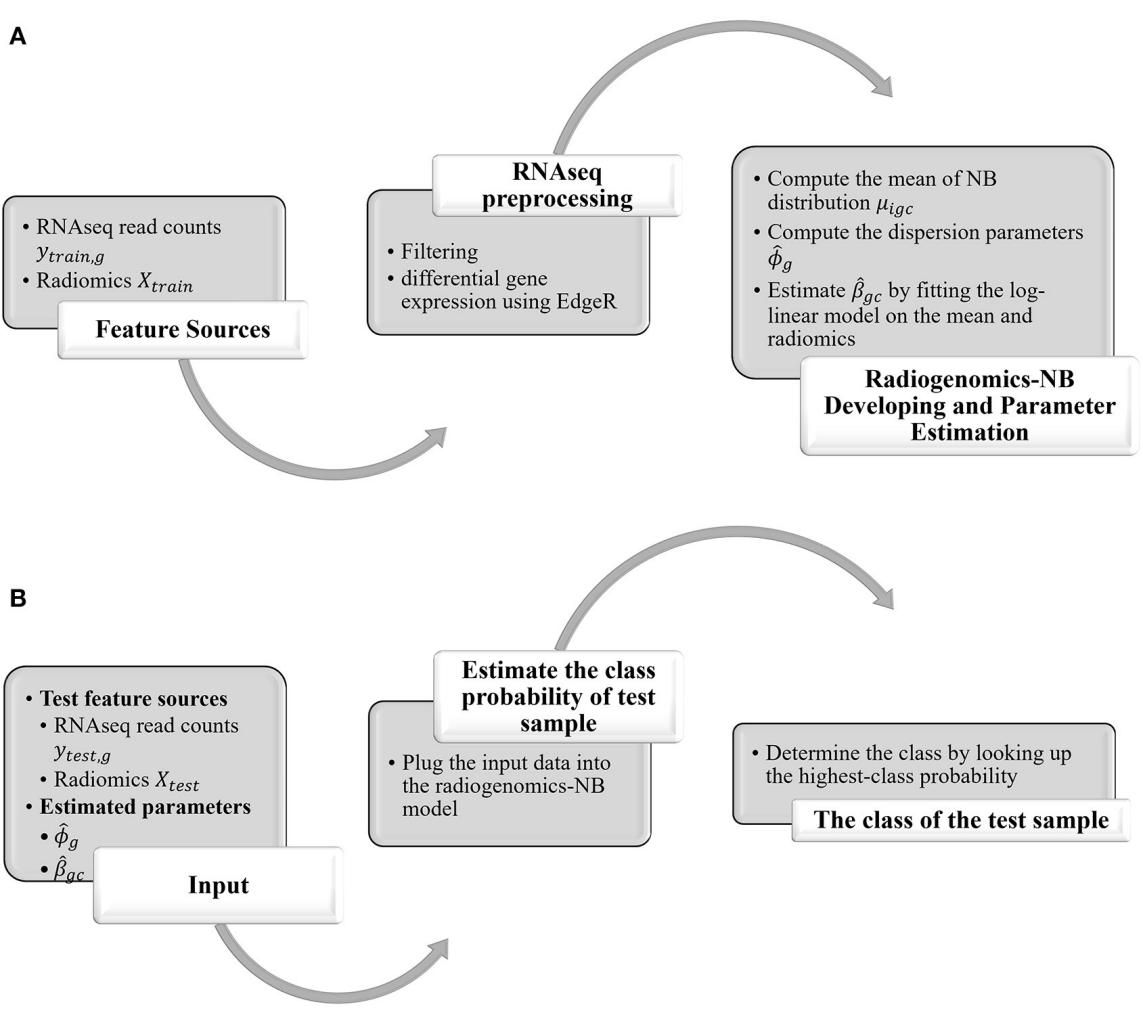
Overall Flow diagram of the proposed radiogenomics-NB prediction model. **(A)** radiogenomics-NB model utilizing the training data. **(B)** class prediction of a test sample using the developed radiogenomics-NB model.

In this study, we propose a radiogenomics-NB method for glioma molecular grading and prediction. Figure 1 illustrates an overall flow diagram of the proposed radiogenomics-NB model. In Figure 1A, we fit the NB distribution to RNAseq read counts of the training dataset and estimate the model mean and dispersion parameter. Then, we use the estimated mean along with the predictor radiomics vector in a log-linear regression model to estimate the model regression coefficients. The dispersion parameter is estimated using the weighted likelihood empirical Bayes method (19). In Figure 1B, the estimated parameters of regression coefficients and the dispersion parameters along with the sample radiomics and its RNAseq read counts are utilized to predict the class label of a future test sample. A complete mathematical derivation of the radiogenomics-NB model is presented in the following subsection.

### Prediction Using Negative Binomial Regression Model

To fuse radiomics with RNAseq read counts data in an NB model, the following parametrization is defined:

Let *C* be the total number of classes, and *I*_*c*_ ∈ (1, …, *n*_*c*_) be the indices of samples in class *c* for *c* = 1, …, *C*. The examples of different classes include:

*IDH* mutated vs. wildtype *IDH* (*C* = 2),

*1p/19q codeletion*: *codeletion* vs. *non-codeletion* (*C* = 2),

Mutated *ATRX* vs. wildtype (*C* = 2).

Let *Y*_*i*_ = (*y*_*i*1_, *y*_*i*2_, …, *y*_*iG*_) be the RNAseq read counts training sample in the class label *c* and *G* is the total number of RNAseq. The purpose of this study is to predict the class label *c* of a future observation *Y*_*t*_ using training samples associated with known class labels: *p* (*c*|*Y*_*t*_)∝*p*(*Y*_*t*_|*c*)*p*_*c*_, where *p*_*c*_ is the probability of class *c*.

Using Bayes' rule, we have,

(1) $$\begin{array}{l} p\left(c|Y_{i}\right)\propto p\left(Y_{i}|c\right)p_{c}; \end{array}$$

where, *p*(*Y*_*i*_| *c*) is the pdf of the sample *Y*_*i*_ in class *c*, and *p*_*c*_ is the prior probability that one sample comes from class *c*. The pdf of class-specific *c* of RNAseq read counts of sample *Y*_*i*_ and of RNAseq *g* is,

(2) $$\begin{array}{l} P\left(Y_{ig}=y_{ig}|c\right)=\frac{\Gamma \left(\phi_{g}^{-1}+y_{ig}\right)}{\Gamma \left(\phi_{g}^{-1}\right)y_{ig}!}{\left(\frac{\phi_{g}\mu_{igc}}{1+\phi_{g}\mu_{igc}}\right)}^{y_{ig}}\\ {\left(\frac{1}{1+\phi_{g}\mu_{igc}}\right)}^{\phi_{g}^{-1}}. \end{array}$$

In this parameterization, *Y*_*ig*_ represents a count response of RNAseq, where μ_*igc*_ represents the mean, ϕ_*g*_ represents the dispersion parameter, *E*(*Y*_*ig*_) = μ_*igc*_, and $Var\left(Y_{ig}\right)=\;\mu_{igc}+\mu_{igc}\phi_{g}^{2}$. Note we assume that all RNAseq are independent of each other, so we have,

(3) $$\begin{array}{l} p\left(Y_{i}|c\right)=\;\overset{G}{\underset{g=1}{\prod }}P\left(Y_{ig}=y_{ig}\right). \end{array}$$

Evaluating Equation (1) requires an estimation of *p*(*Y*_*i*_|*c*) and *p*_*c*_. The model in Equation (2) states that *Y*_*ig*_ ~ *NB*(μ_*igc*_, ϕ_*g*_). We first estimate ϕ_1_, ϕ_2_, …, ϕ_*G*_, and μ_*i*1*c*_, μ_*i*2*c*_, …, μ_*iGc*_ of all the training samples *n*_*c*_, and all RNAseq *G*. The mean is estimated as μ_*igc*_ = *s*_*ic*_λ_*gc*_, where *s*_*ic*_ is the size factor (26, 27) which is used to scale RNAseq counts for the *ith* sample (in class *c*), λ_*gc*_ is the total number of reads of RNAseq *g* across all samples in class *c*. For prior *p*_*c*_, we assume all classes are equally likely, *p*_*c*_ = 1/*C*. Note that μ_*igc*_, *s*_*ic*_, *and λ*_*gc*_ are estimated for each class *c*.

Next, plugging these estimates into Equation (2) and using the assumption of independent RNAseq, Equation (1) yields,

(4) $$\begin{array}{l} \text{log}(p(c|Y_{i}))\;=\text{ log}(p(Y_{i}|c)\;+\text{ log}(p_{c}). \end{array}$$

The log-likelihood log(*p*(*Y*_*i*_|*c*)) is written as,

(5) $$\begin{array}{l} \;\log\left(p\left(Y_{i}|c\right)\right)=\log\left(\prod_{g=1}^{G}P\left(Y_{ig}=y_{ig}|c\right)\right)\\ =\log\left(\prod_{g=1}^{G}\frac{\Gamma \left(\phi_{g}^{-1}+y_{ig}\right)}{\Gamma \left(\phi_{g}^{-1}\right)y_{ig}!}{\times \left(\frac{\phi_{g}\mu_{igc}}{1+\phi_{g}\mu_{igc}}\right)}^{y_{ig}}\times {\left(\frac{1}{1+\phi_{g}\mu_{igc}}\right)}^{\phi_{g}^{-1}}\right). \end{array}$$

Equation (5) can be written as,

(6) $$\begin{array}{l} log\left(p\left(Y_{i}|c\right)\right)=\;\sum_{g=1}^{G}{log\left(\frac{\phi_{g}\mu_{igc}}{1+\phi_{g}\mu_{igc}}\right)}^{y_{ig}}\\ \;+\sum_{g=1}^{G}{log\left(\frac{1}{1+\phi_{g}\mu_{igc}}\right)}^{\phi_{g}^{-1}}\\ \;+\sum_{g=1}^{G}log\left(\frac{\Gamma \left(\phi_{g}^{-1}+y_{ig}\right)}{\Gamma \left(\phi_{g}^{-1}\right)y_{ig}!}\right). \end{array}$$

Rewriting Equation (6) yields,

(7) $$\begin{array}{l} \log\left(p\left(Y_{i}|c\right)\right)=\;\overset{G}{\underset{g=1}{\sum }}y_{ig}\log\left(\phi_{g}\mu_{igc}\right)\;-\;\overset{G}{\underset{g=1}{\sum }}y_{ig}\log\left(1+\phi_{g}\mu_{igc}\right)\\ \;-\overset{G}{\underset{g=1}{\sum }}\frac{1}{\phi_{g}}\log\left(1+\phi_{g}\mu_{igc}\right)\\ \;+\;\overset{G}{\underset{g=1}{\sum }}\log\left(\frac{\Gamma \left(\phi_{g}^{-1}+y_{ig}\right)}{\Gamma \left(\phi_{g}^{-1}\right)y_{ig}!}\right). \end{array}$$

The proposed NB model of genomics relates to the radiomics (imaging features) **X** through the mean parameters μ_*igc*_ (estimated mean of an *ith* sample and RNAseq *g* in class *c*). We assume a log-linear regression model for estimating the mean μ_*igc*_ in terms of the radiomics (imaging features) is given as follows:

(8.a) $$\begin{array}{l} \log\left(\mu_{igc}\right)=\;X_{i}\beta_{gc}; \end{array}$$

(8.b) $$\begin{array}{l} \log\left(s_{ic}\lambda_{gc}\right)=\;X_{i}\beta_{gc}; \end{array}$$

where *X*_*i*_ is a *p*-dimensional of radiomics, β_*gc*_ is a *p*-dimensional vector of unknown regression coefficients (translate the relationship between *X* and *Y* through μ_*igc*_). The estimation of β_*gc*_ depends on class *c* and gene *g* of the *ith* sample. Hence, if there are two classes, we will need to estimate β_*g*1_ and β_*g*2_ (one from each class).

Plugging Equations (8.a) into Equation (7), yields,

(9) $$\begin{array}{l} \log\left(p\left(Y_{i}|c\right)\right)=\;\sum_{g=1}^{G}y_{ig}\log\left(\phi_{g}\exp\left(X_{i}\beta_{gc}\right)\right)\\ \;-\;\sum_{g=1}^{G}y_{ig}\log\left(1+\phi_{g}\exp\left(X_{i}\beta_{gc}\right)\right)\\ \;-\sum_{g=1}^{G}\frac{1}{\phi_{g}}\log\left(1+\phi_{g}\exp\left(X_{i}\beta_{gc}\right)\right)\\ \;+\sum_{g=1}^{G}log\left(\frac{\Gamma \left(\phi_{g}^{-1}+y_{ig}\right)}{\Gamma \left(\phi_{g}^{-1}\right)y_{ig}!}\right). \end{array}$$

Using the estimated ${\hat{\beta }}_{gc}$, and ${\hat{\phi }}_{g}$ from the *training* data, we classify a *test* observation *Y*_*t*_ as follows,

(10) $$\begin{array}{l} \text{log}(p(c|Y_{t}))\;=\text{ log}(p\left(Y_{t}|c\right)+\;log\left(p_{c}\right); \end{array}$$

and,

(11) $$\begin{array}{l} log\left(p\left(c|Y_{t}\right)\right)=\;\sum_{g=1}^{G}y_{tg}\;log\left({\hat{\phi }}_{g}\exp\left(X_{t}{\hat{\beta }}_{gc}\right)\right)\\ \;-\;\sum_{g=1}^{G}y_{tg}\;log\left(1+{\hat{\phi }}_{g}\exp\left(X_{t}{\hat{\beta }}_{gc}\right)\right)\\ \;-\sum_{g=1}^{G}\frac{1}{\phi_{g}}\;log\left(1+{\hat{\phi }}_{g}\exp\left(X_{t}{\hat{\beta }}_{gc}\right)\right)\\ \;+\sum_{g=1}^{G}log\left(\frac{\Gamma \left({\hat{\phi }}_{g}^{-1}+y_{tg}\right)}{\Gamma \left({\hat{\phi }}_{g}^{-1}\right)y_{tg}!}\right)+\;log\left(p_{c}\right). \end{array}$$

### Radiogenomics-NB Model Parameter Estimation

#### Estimating Dispersion ϕ_g_ Using Weighted Likelihood Empirical Bayes

Various methods for estimating the dispersion parameter are proposed in the literature. The EdgeR method applies a weighted conditional log-likelihood method to estimate the dispersion parameter (19). The weighted conditional log-likelihood (WL) for ϕ_*g*_ is defined as a weighted combination of the individual (per-gene) likelihood *l*_*g*_(ϕ_*g*_) and common *l*_*C*_(ϕ_*g*_) likelihood:

(12) $$\begin{array}{l} WL\left({\hat{\phi }}_{g}\right)=\;l_{g}\left(\phi_{g}\right)\;+\;\alpha l_{C}\left(\phi_{g}\right); \end{array}$$

where α is the weight of *l*_*C*_(ϕ_*g*_).

In EdgeR, ${\hat{\phi }}_{g}$ is assumed to be normally distributed with means ϕ_*g*_ and known variance τ^2^, and has the following hierarchical model:

(13) $$\begin{array}{l} {\hat{\phi }}_{g}|\phi_{g}\sim N\left(\phi_{g},\tau^{2}\right),\;and\;\phi_{g}\sim N\left(\phi_{0},\tau_{0}^{2}\right). \end{array}$$

Under this hierarchical normal model, the maximum weighted conditional log-likelihood estimator is given as:

(14) $$\begin{array}{l} {\hat{\phi }}_{g}^{WL}=\frac{{\hat{\phi }}_{g}/\tau^{2}\;+\alpha \sum_{i=1}^{G}{\hat{\phi }}_{i}/\tau_{i}^{2}\;}{1/\tau^{2}\;+\alpha \sum_{i=1}^{G}1/\tau_{i}^{2}}; \end{array}$$

where,

(15) $$\begin{array}{l} 1/\alpha =\overset{G}{\underset{i=1}{\sum }}\tau_{0}^{2}/\tau_{i}^{2} \end{array}$$

and,

(16) $$\begin{array}{l} \phi_{0}\;={\hat{\phi }}_{0}=\;\frac{\sum_{i=1}^{G}{\hat{\phi }}_{i}/\tau_{i}^{2}}{\sum_{i=1}^{G}1/\tau_{i}^{2}}. \end{array}$$

#### Computation of the Mean of RNAseq μ_igc_

The size factor *s*_*ic*_ of sample *i* and class *c* is the total number of RNAseq read counts of that sample divided by the total number of all RNAseq read counts across all training samples (in class *c*). The size factor estimation is vital to account for the different sequencing depth (library size) that may be used to sequence different samples and is computed as follows:

(17) $$\begin{array}{l} s_{ic}\;=\;\frac{\sum_{g=1}^{G}y_{igc}}{\sum_{i=1}^{n_{c}}\sum_{g=1}^{G}y_{igc}}; \end{array}$$

where, *y*_*igc*_ is the RNAseq read count of sample *i* and RNAseq *g* in class *c*, and *n*_*c*_ is the total number of samples in class *c*.

The mean μ_*igc*_ of sample *i* and RNAseq *g* in class *c* is then estimated as μ_*igc*_ = *s*_*ic*_λ_*gc*_, where λ_*gc*_ is the total number of reads per RNAseq in class *c*, and is computed as follows:

(18) $$\begin{array}{l} \lambda_{gc}\;=\overset{N_{c}}{\underset{i=1}{\sum }}y_{igc}. \end{array}$$

Using the estimated value of μ_*igc*_, the values of β_*gc*_ are computed using equation 8.a as follows:

(19) $$\begin{array}{l} \beta_{gc}=X_{i}\log\left(\frac{\sum_{g=1}^{G}y_{igc}}{\sum_{i=1}^{n_{c}}\sum_{g=1}^{G}y_{igc}}\sum_{i=1}^{N_{c}}y_{igc}\right). \end{array}$$

**Figure 2:**
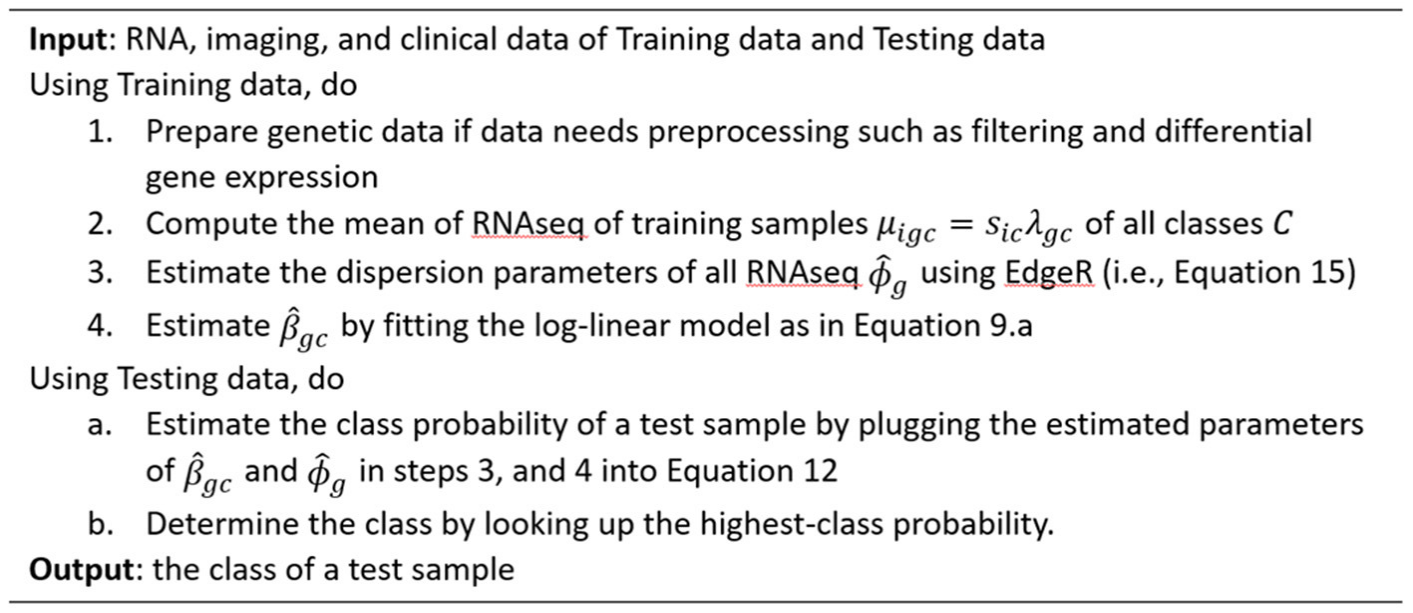
Algorithm of prediction using radiogenomics Negative Binomial classification model.

The algorithm in Figure 2 illustrates the steps of estimating the different parameters in the radiogenomics-NB classification model.

## Experimental Results

### Dataset

The dataset in this study consists of 108 pre-operative lower grade glioma (LGG) patients that are described in Menze et al. (28), Bakas et al. (29), and Bakas S. et al. (30). Four sequences of the MRI are provided with the dataset: pre-contrast T1-weighted (T1), post-contrast T1-weighted (T1Gd), T2-weighted (T2), and T2 Fluid Attenuated Inversion Recovery (FLAIR). These scans are skull-stripped, re-sampled to 1 *mm*^3^ resolution, and co-registered to the T1 template. The dataset provides the segmented sub-regions of the LGG: Gadolinium enhancing tumor (ET), the peritumoral edema (ED), and necrosis along with non-enhancing tumor (NCR/NET).

RNAseq read counts data (with a total number of 56830 RNAseq), molecular alterations (*IDH* mutation, *1p/19q codeletion*, and *ATRX*), grade (II and III), and the clinical dataset can be found and downloaded from The Cancer Genome Atlas (TCGA) dataset in the Genomic Data Commons (GDC) Data Portal (https://portal.gdc.cancer.gov/). RNAseq are primarily obtained from solid portions of tumor. The clinical dataset is de-identified in compliance with the Health Insurance Portability and Accountability Act of 1996 (HIPAA). The distribution of the data is as follows: (i) *IDH* mutation: 85 Mutant and 23 wildtype (WT), (ii) *1p/19q codeletion*: 27 *codeletion* and 81 *non-codeletion*, and (iii) *ATRX* status: 43 Mutant and 65 WT. The range of the patients' age at diagnosis is 20–75 years, and the median age is 46.5 years.

### Data Preparation

In this study we first filter RNAseq read counts to remove RNAseq with very low value of read counts before performing any statistical analysis. RNAseq with very low read counts hold very little information because an RNAseq of biological importance needs to be expressed at some minimal level. We utilize a quantile filter (31) with a quantile threshold of 0.25. This step returns each RNAseq that has a mean across all samples higher than the defined quantile threshold of 0.25. Then, we reduce the number of RNAseq that are used in the radiogenomics-NB models, by utilizing EdgeR (18–20) to extract the differentially expressed RNAseq (DERs). DERs reflect the significance of a gene in a certain biological condition. In this study, we select the top 10, 20, 30, 50, 100, and 150 DERs (see Supplementary Table 1).

**Figure 3:**
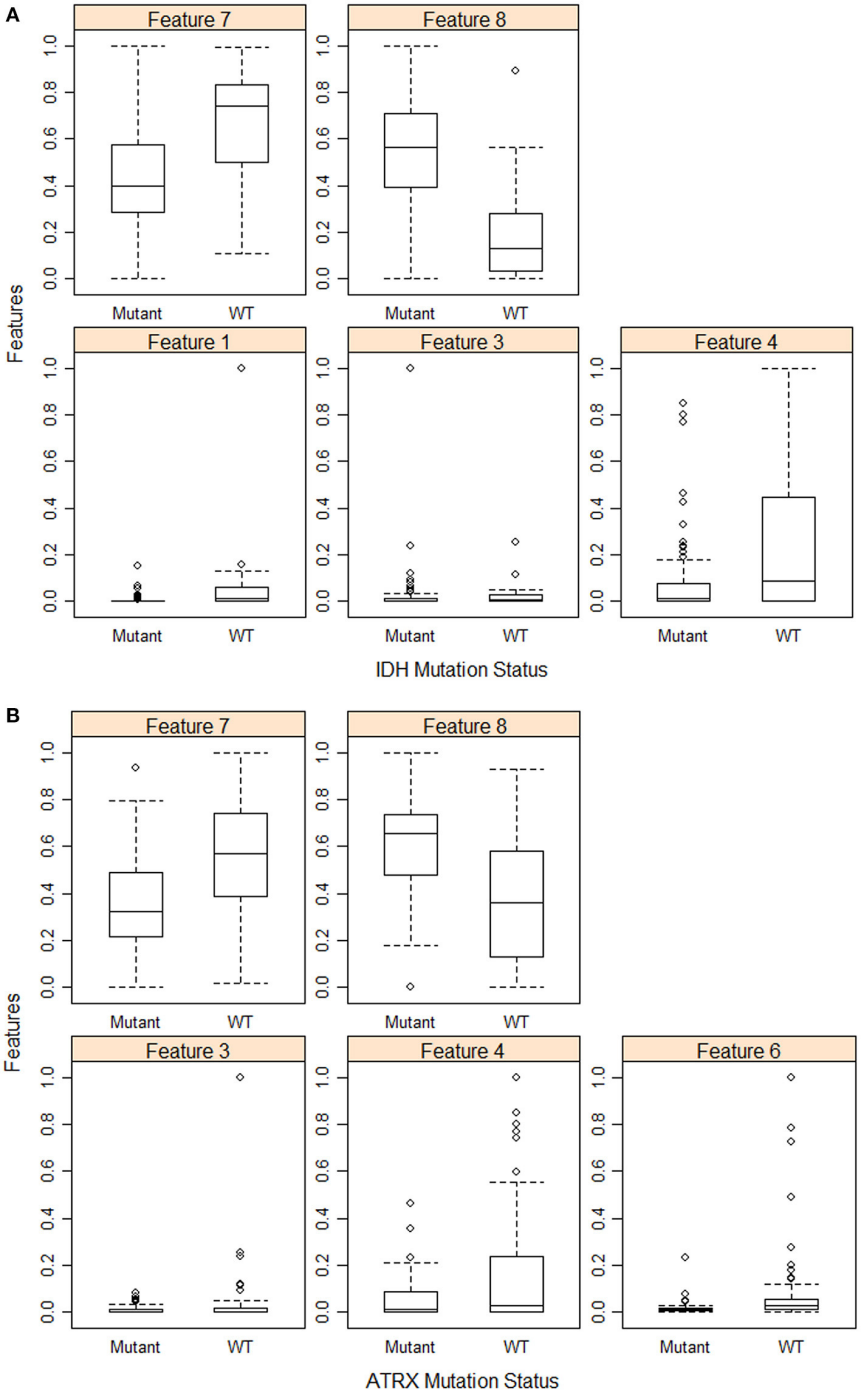
Feature distribution plot of the significant volumetric radiomic associated with **(A)** *IDH* mutations, and **(B)** *ATRX* mutations.

Furthermore, we use eight volumetric radiomics features as illustrated in Table 1. ANOVA analysis for radiomics in Table 1 shows that feature numbers 1, 3, 4, 7, and 8 are significantly associated (ANOVA test, *p* < 0.05) with *IDH* mutations as illustrated in Figure 3A. Our analysis also indicates that feature number 2 is marginally associated (ANOVA test, *p* = 0.07) with *1p/19q codeletion*. Furthermore, our analysis indicates that feature numbers 3, 4, 6, 7, and 8 are significantly associated (ANOVA test, *p* < 0.05) with *ATRX* mutations as illustrated in Figure 3B. Additionally, our analysis reveals that thresholding feature number 6 around the mean creates an ordinal feature that is significantly associated (ANOVA test, *p* < 0.05) with *IDH* mutations, *1p/19q codeletion*, and *ATRX* mutations. Likewise, thresholding feature numbers 1, 3, 5, 7, and 8 around their means converts these features into ordinal features that are significantly associated (ANOVA test, *p* < 0.05) with *IDH* and *ATRX* mutations. Moreover, thresholding feature numbers 5, 6, 7, and 8 around their median converts these features into ordinal features that are significantly associated (ANOVA test, *p* < 0.05) with *IDH* and *ATRX* mutations.

**Table 1:** Radiomics features description and their ANOVA *p-value* association with *IDH* mutations, *1p/19q codeletion*, and *ATRX* mutations.

**Feature number**	**Feature description**	***p-value* of *IDH* mutation**	***p-value* of *1p/19q codeletion***	***p-value* of *ATRX* mutation**
1	the size of the enhancing tumor to the necrosis size	<0.005	0.393	0.178
2	the size of the enhancing tumor to the size of enhancing tumor and necrosis	0.8630	0.070	0.239
3	the size of the enhancing tumor to the edema size	<0.005	0.600	<0.005
4	the size of the enhancing tumor to the whole tumor size	<0.005	0.707	0.027
5	the size of the edema to the necrosis size	0.188	0.996	0.114
6	the size of the edema to the size of enhancing tumor and necrosis	0.138	0.789	0.0237
7	the size of the edema to the whole tumor size	<0.005	0.131	<0.005
8	and the size of the necrosis to the whole tumor size	<0.005	0.221	<0.005

Few other studies suggest that these volumetric imaging features and their ratios are associated with and predictive of several mutations in gliomas (32–35).

The 108 LGG cases are randomly split into 80% training and 20% testing sets, and a balanced distribution of the target molecular alteration is ensured in the training and testing sets in each molecular classifier. The trained model classifier is developed using the training set. Model performance prediction is estimated and reported using the testing sets in terms of accuracy, balanced accuracy, F1 score, sensitivity, specificity, negative predictive value, and positive predictive value. The training set is utilized to build our radiogenomics-NB classifier as shown in steps **1-4** in Figure 2. The testing set is used to estimate the performance of the classifier as shown in steps **a** and **b** in Figure 2. Authors In Dong et al. (23), Maufroy et al. (36), Pan et al. (37), and Vabalas et al. (38) repeat training and testing analysis for a specific number of times to ensure the robustness of the model performance. Consequently, in this work, we repeat the whole procedure 100 times independently for the 3 molecular alterations and then report the mean and standard deviation of the classifiers' performance using the testing sets.

**Figure 4:**
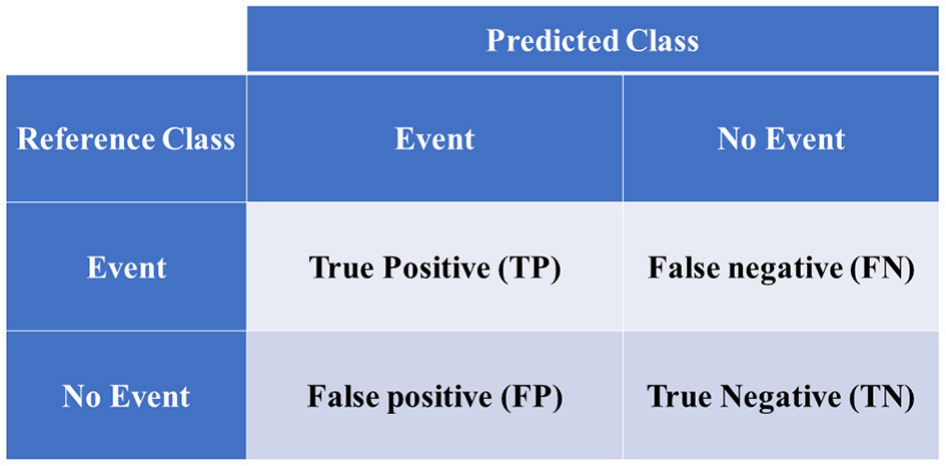
Confusion matrix of binary classification.

Model performance parameters are computed based on the confusion matrix in Figure 4 as follows:

(20) $$\begin{array}{l} Accuracy\;=\;TP\;+\frac{TN}{TP}+\;TN\;+\;FP\;+\;TN; \end{array}$$

(21) $$\begin{array}{l} Sensitivity\;=\frac{TP}{TP}+\;FN; \end{array}$$

(22) $$\begin{array}{l} Specificity\;=\frac{TN}{FP}+TN; \end{array}$$

(23) $$\begin{array}{l} Positive\;predictive\;value\;=\frac{TP}{TP}+FP; \end{array}$$

(24) $$\begin{array}{l} Negative\;predictive\;value\;=\frac{TN}{FN}+TN; \end{array}$$

(25) $$\begin{array}{l} Balanced\;Accuracy\;=\frac{Sensitivity\;+\;Specificity}{2},\text{and} \end{array}$$

(26) $$\begin{array}{l} F1\;score\;=TP\left(TP\;+\frac{FP+FN}{2}\right); \end{array}$$

where TP is the true positive, TN is the true negative, FP is the false positive, and TN is the true negative.

### Radiogenomics-NB Models Using Different Number of Differentially Expressed RNAs

**Figure 5:**
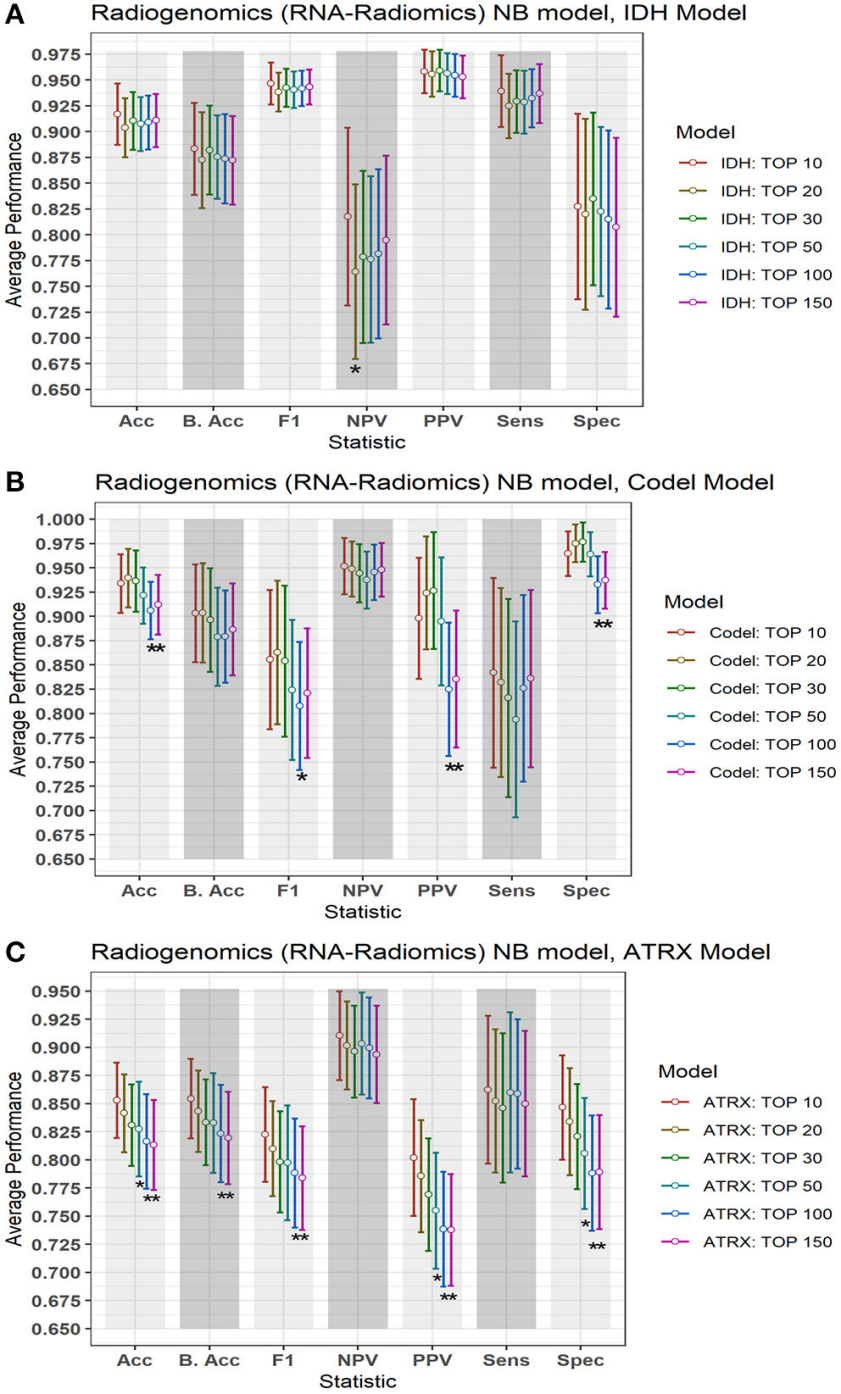
Performance of the proposed radiogenomics-NB model using a different number of DERs. **(A)** Radiogenomics-NB *IDH*, **(B)** Radiogenomics-NB *Codeletion*, and **(C)** Radiogenomics-NB *ATRX* models. The average performance (of the Acc, B. Acc, F1, NPV, PPV, Sens, and Spec) is computed across 100 testing sets/splits. Y-axis represents the average performance of the different statistics on the X-axis. Different colors represent the radiogenomics-NB model with different numbers of DERs. The error bar represents one standard deviation. Asterisk “^*^” represents a statically significant difference between the performance achieved when using the top 10 DERs (in red) and using the number of DER where the star is located.

In this section, we investigate the importance of using different numbers of DERs on the performance of the radiogenomics-NB model. LGG radiogenomics-NB mutation prediction models are developed based on the top 10, 20, 30, 50, 100, and 150 DERs. The performance of the radiogenomics-NB *IDH* model using the top 10 DERs achieves slightly higher performance. However, such improvement is not statistically significant (ANOVA test, *p* > 0.05) when compared to the performance of the *IDH* models with the other number of DERs (Figure 5A) except for negative predictive value (NPV) performance when using the top 20 DERs. Using the top 20 DERs in the *IDH* model achieves significantly worse NPV when compared to the NPV achieved using the top 10 DERs (ANOVA test, *p* < 0.05). Radiogenomics-NB *IDH* model with the top 10 DERs (red line in Figure 5A) achieves an overall accuracy (Acc) of 0.92 ± 0.06, sensitivity (Sens) of 0.94 ± 0.07, specificity (Spec) of 0.83 ± 0.18, positive predictive value (PPV) of 0.96 ± 0.04, negative predictive value (NPV) of 0.82 ± 0.17, F1 score of 0.95 ± 0.04, and balanced accuracy (B. Acc) of 0.88 ± 0.09, respectively.

Radiogenomics-NB *codeletion* models achieve similar performance (ANOVA test, *p* > 0.05) using the top 10, 20, 30, and 50 DERs as shown in Figure 5B. Furthermore, using the top 100 and 150 DERs in the *codeletion* model achieves significantly worse performance when compared to the performance of using the top 10 DERs (ANOVA test, *p* < 0.05). Using the top 10 DERs, the radiogenomics-NB *codeletion* model achieves an accuracy of 0.93 ± 0.06, a balanced accuracy of 0.90 ± 0.10, F1 score of 0.86 ± 0.14, a sensitivity of 0.84 ± 0.19, a specificity of 0.96 ± 0.04, an NPV of 0.95 ± 0.06, and a PPV of 0.90 ± 0.12, respectively.

Radiogenomics-NB *ATRX* model also achieves similar performance (ANOVA test, *p* > 0.05) using the top 10, 20, and 30 DERs, even though the performance when using the top 10 DERs is slightly better as illustrated in Figure 5C. Using the top 10 DERs, the *ATRX* model achieves an accuracy of 0.85 ± 0.07, a balanced accuracy of 0.85 ± 0.07, an F1 score of 0.82 ± 0.08, a sensitivity of 0.86 ± 0.13, a specificity of 0.85 ± 0.09, an NPV of 0.91 ± 0.08, and a PPV of 0.80 ± 0.10, respectively.

### Comparative Analysis

**Figure 6:**
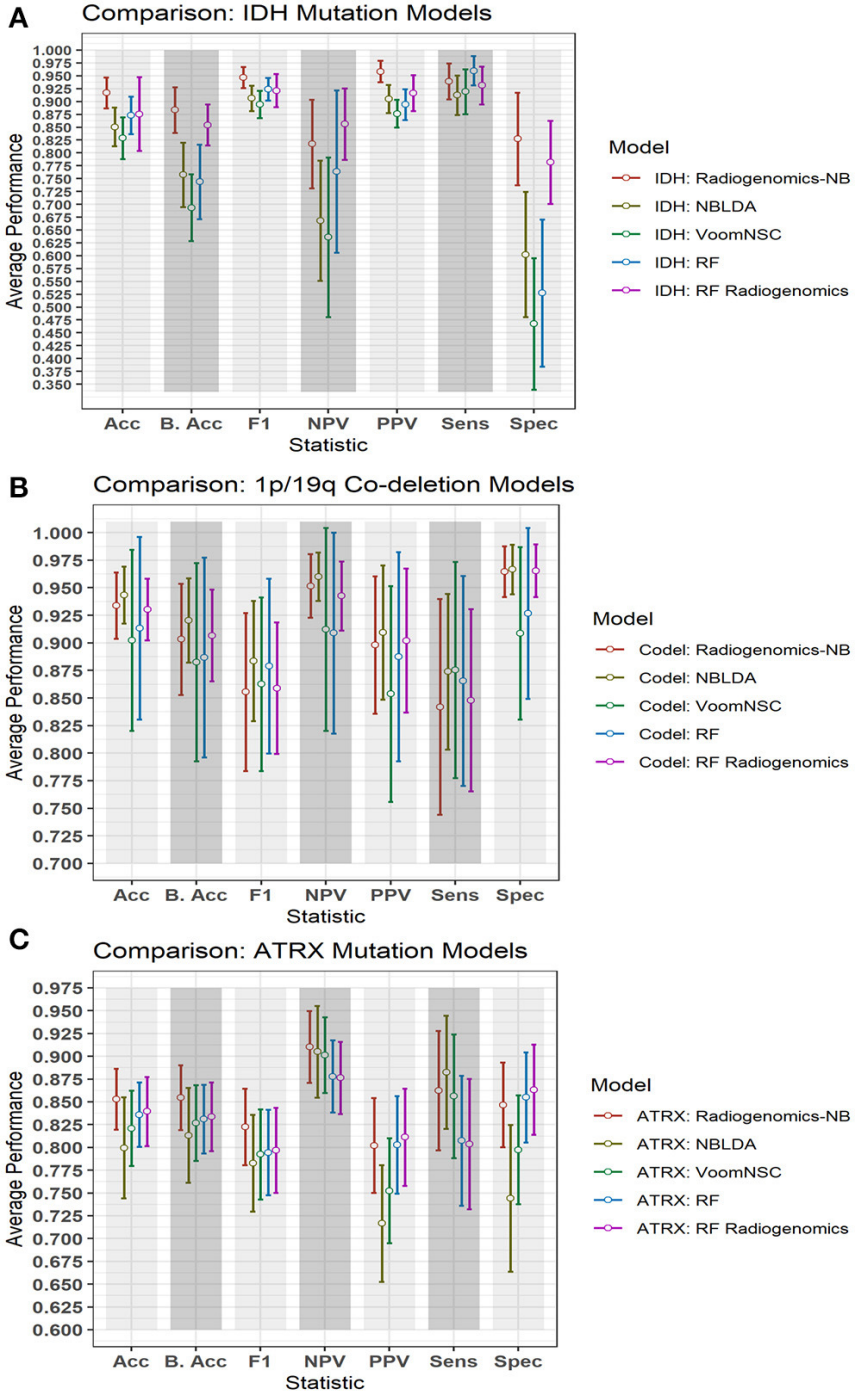
Comparison of performance between our radiogenomics-NB model and different classifiers. The comparison is performed using the **(A)** *IDH* mutations, **(B)** *1p/19q codeletion*, and **(C)** *ATRX* mutations dataset. The average performance (of the Acc, B. Acc, F1, NPV, PPV, Sens, and Spec) is computed across 100 test sets. The error bar represents one standard deviation. RNAseq that are used in developing all classifiers represent the top 10 DERs in the training sets between mutated and WT *IDH* group, codeleted and non-codeleted groups, and mutated and WT *ATRX* mutation, respectively. *Y*-axis represents the average performance of the different statistics on the *X*-axis. Different colors represent different classifiers.

Figure 6 illustrates a graphical performance comparison between our radiogenomics-NB model with that of four different classifiers in the literature: NBLDA (23), VoomNSC (12, 13), RF-genomics where we first log-transformed (20) the RNAseq into a normal distribution, and RF-radiogenomics. Note that the number of DERs that we apply to develop these classifiers is 10 DERs. Moreover, when developing these classifiers, the 108 LGG cases are randomly split into 80% training and 20% testing sets, and balanced distribution is ensured when developing the different classifiers. The trained model classifier is developed using the training set, and 10-fold cross-validation is performed to identify the tuning parameters in the different classifiers. Model performance prediction is estimated and reported using the testing sets. Additionally, to ensure the robustness of the different classifiers' performance, we repeat the whole procedure 100 times independently and every training/testing set is utilized to develop and estimate the performance of each classifier.

The NBLDA (23) classifier is developed by fitting NB to the top 10 DERs; then the mean and dispersion parameter are estimated from these DERs. In RF-genomics, the top 10 DERs of the training sets are first log-transformed into normal distribution and then fed into RF to build the RF-genomics classifier. In RF-radiogenomics, radiomics (eight volumetric features described previously in section Data Preparation) are utilized with the log-transformed DERs and then fed into RF to build the RF-radiogenomics classifier. VoomNSC (12, 24) is developed by first applying the Voom-based transformation on the 10 DERs and then applying the NSC classifier as illustrated in Zararsiz et al. (12) and Tibshirani et al. (24).

Comparing the performance of our radiogenomics-NB *IDH* model with that of NBLDA, RF-genomics, and VoomNSC, the radiogenomics-NB *IDH* significantly outperforms (ANOVA test, *p* < 0.05) these methods as shown in Figure 6A and Table 2. Additionally, our radiogenomics-NB *IDH* model significantly outperforms (ANOVA test, *p* < 0.05) the F1 score, balanced accuracy, and PPV performance of the RF-radiogenomics method whereas it achieves a similar (ANOVA test, *p* > 0.05) accuracy, sensitivity, and specificity. Our radiogenomics-NB *IDH* model archives an accuracy of 0.92 ± 0.06, a sensitivity of 0.94 ± 0.07, a specificity of 0.93 ± 0.18, an F1 score of 0.95 ± 0.04, and a balanced accuracy of 0.88 ± 0.09, respectively. The RF-radiogenomics-*IDH* model achieves an accuracy of 0.88 ± 0.17, a sensitivity of 0.93 ± 0.07, a specificity of 0.78 ± 0.16, an F1 score of 0.92 ± 0.06, and a balanced accuracy of 0.85 ± 0.08, respectively.

**Table 2:** Probability of significant difference using ANOVA test between the differentially expressed radiogenomics-NB model and different classifiers using the *IDH* dataset.

***IDH***	**Accuracy**	**Sensitivity**	**Specificity**	**PPV**	**NPV**	**F1**	**Balanced accuracy**
radiogenomics-NB vs. NBLDA	**0.000**	**0.010**	**0.000**	**0.000**	**0.000**	**0.000**	**0.000**
radiogenomics-NB vs. VoomNSC	**0.000**	0.075	**0.000**	**0.000**	**0.000**	**0.000**	**0.000**
radiogenomics-NB vs. RF	**0.001**	0.023	**0.000**	**0.000**	0.138	**0.000**	**0.000**
radiogenomics-NB vs. RF-radiogenomics	0.069	0.432	0.061	**0.000**	0.084	**0.001**	**0.01**

*A statistically significant difference exists if p < 0.05. Values in bold show a significant improvement of our radiogenomics-NB IDH over the compared one*.

Our radiogenomics-NB *codeletion* model (Figure 6B and Table 3) performance is similar to NBLDA, RF-genomics, VoomNSC, and RF-radiogenomics models, except for the specificity and NPV performance when using RF-genomics and VoomNSC. The specificity and NPV of our model are significantly higher than those achieved by RF-genomics and VoomNSC. Our radiogenomics-NB *codeletion* model achieves an accuracy of 0.93 ± 0.06, a sensitivity of 0.84 ± 0.20, a specificity of 0.96 ± 0.5, an F1 score of 0.86 ± 0.14, and a balanced accuracy of 0.90 ± 0.10, respectively.

**Table 3:** Probability of significant difference using ANOVA test between the differentially expressed radiogenomics-NB model and different models using the *1p/19q codeletion* dataset.

**CODEL**	**Accuracy**	**Sensitivity**	**Specificity**	**PPV**	**NPV**	**F1**	**Balanced accuracy**
radiogenomics-NB vs. NBLDA	0.232	0.186	0.756	0.514	0.253	0.123	0.181
radiogenomics-NB vs. VoomNSC	0.072	0.228	**0.001**	0.057	**0.042**	0.742	0.317
radiogenomics-NB vs. RF	0.242	0.390	**0.020**	0.636	**0.027**	0.271	0.42
radiogenomics-NB vs. RF-radiogenomics	0.671	0.815	0.893	0.825	0.282	0.855	0.792

*A statistically significant difference exists if p < 0.05. Values in bold show a significant improvement of our radiogenomics-NB codeletion over the compared one*.

The performance of our radiogenomics-NB *ATRX* model as shown in Figure 6C and Table 4 outperforms both NBLDA and VoomNSC significantly (ANOVA test, *p* < 0.05). However, comparing our *ATRX* model to RF-genomics, our model achieves significantly better balanced-accuracy, F1 score, NPV, and sensitivity. Additionally, comparing our *ATRX* model to RF-radiogenomics, our model achieves significantly (ANOVA test, *p* < 0.05) better sensitivity but achieves similar accuracy, balanced-accuracy, F1 score, and sensitivity. Our radiogenomics-NB *ATRX* model achieves an accuracy of 0.85 ± 0.07, a sensitivity of 0.86 ± 0.13, a specificity of 0.85 ± 0.09, an F1 score of 0.82 ± 0.08, and a balanced accuracy of 0.85 ± 0.07, respectively. The RF-radiogenomics *ATRX* model achieves an accuracy of 0.84 ± 0.08, a sensitivity of 0.80 ± 0.14, a specificity of 0.86 ± 0.10, an F1 score of 0.80 ± 0.09, and a balanced accuracy of 0.83 ± 0.08, respectively.

**Table 4:** Probability of significant difference using ANOVA test between the differentially expressed radiogenomics-NB model and different models using the *ATRX* dataset.

***ATRX***	**Accuracy**	**Sensitivity**	**Specificity**	**PPV**	**NPV**	**F1**	**Balanced accuracy**
Radiogenomics-NB vs. NBLDA	**0.000**	0.269	**0.000**	**0.000**	0.677	**0.004**	**0.001**
Radiogenomics-NB vs. VoomNSC	**0.003**	0.741	**0.001**	**0.002**	0.432	**0.021**	**0.012**
Radiogenomics-NB vs. RF	0.083	**0.005**	0.540	0.960	**0.004**	**0.026**	**0.025**
Radiogenomics-NB vs. RF-radiogenomics	0.183	**0.003**	0.215	0.561	**0.003**	0.052	0.053

*A statistically significant difference exists if p < 0.05. Values in bold show a significant improvement of our radiogenomics-NB ATRX over the compared one*.

### Gender–Specific Effect Analysis of Radiogenomics-NB

In our LGG dataset, *IDH* mutated patients, unlike *IDH* WT patients, have significantly longer survival (65.7 vs. 19.9 months, log-rank test *p* = 0.004). The association between *IDH* status and overall survival remains significant after stratifying for gender (likelihood ratio test *p* = 0.015). However, the association between *1p/19q codeletion* and *ATRX* status and overall survival is not significant. Additionally, the chi-square test shows no significant association (*p* > 0.05) between gender and *IDH* status, *1p/19q codeletion*, and *ATRX* status. Table 5 shows patient *IDH* status, *1p/19q codeletion*, and *ATRX* status distribution based on gender.

**Table 5:** Gender-based distribution of *IDH* status, *1p/19q codeletion*, and *ATRX* status in the LGG dataset.

	***IDH*** **status**		**1p/19q codeletion**		***ATRX*** **status**	
	**Mutant**	**WT**	**Codeletion**	**Non-codeletion**	**Mutant**	**WT**
Female	43	14	14	43	24	33
Male	42	9	13	38	19	32

**Figure 7:**
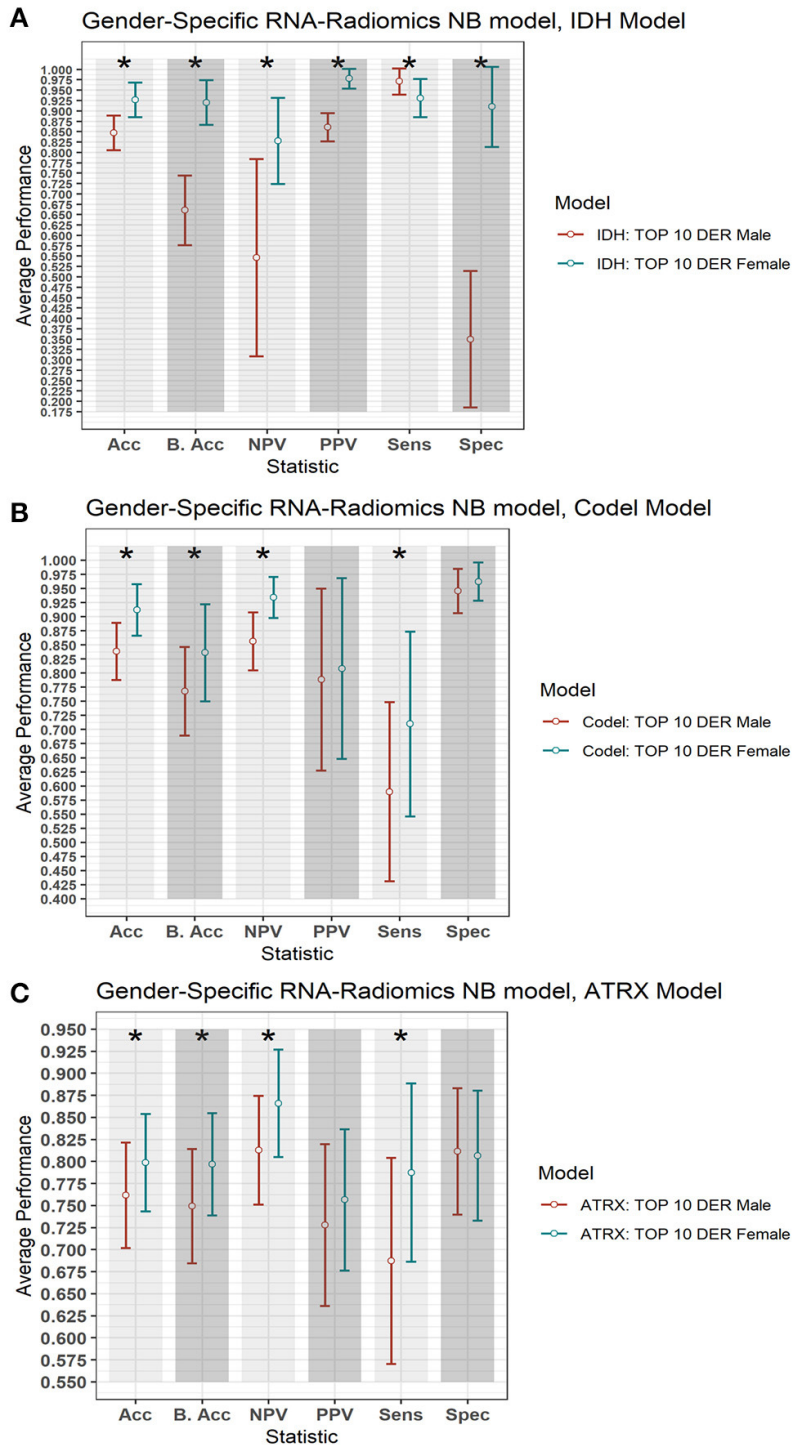
Gender-based radiogenomics-NB models performance. **(A)** *IDH* mutations, **(B)** *1p/19q codeletion*, and **(C)** *ATRX* mutations which are computed across 100 testing sets. The error bar represents one standard deviation. The asterisk ^*^ illustrates a significant difference between the two measurements. *Y*-axis represents the average performance of the different statistics on the *X*-axis. Different colors represent the female- and male-specific radiogenomics-NB models.

To explore the gender-specific effect in the performance of the radiogenomics-NB, we build two radiogenomics-NB models based on gender; male-specific radiogenomics-NB and female-specific radiogenomics-NB. Our analysis indicates that female-specific models significantly outperform (ANOVA test, *p* < 0.05) male-specific models as illustrated in Figure 7. In the radiogenomics-NB *IDH*, female-specific model achieves an accuracy of 0.93 ± 0.08, a sensitivity of 0.93 ± 0.09, a specificity of 0.91 ± 0.10, a PPV of 0.97 ± 0.05, an NPV of 0.83 ± 0.21, and a balanced accuracy of 0.92 ± 0.11, respectively. The male specific *IDH* model achieves an accuracy of 0.85 ± 0.08, a sensitivity of 0.97 ± 0.06, a specificity of 0.35 ± 0.33, a PPV of 0.86 ± 0.07, an NPV of 0.55 ± 0.48, and a balanced accuracy of 0.66 ± 0.17, respectively.

In the radiogenomics-NB *codeletion*, female-specific model achieves an accuracy of 0.91 ± 0.09, a sensitivity of 0.77 ± 0.31, a specificity of 0.96 ± 0.07, a PPV of 0.80 ± 0.32, an NPV of 0.93 ± 0.07, and a balanced accuracy of 0.84 ± 0.17, respectively. The male specific *codeletion* model achieves an accuracy of 0.84 ± 0.10, a sensitivity of 0.56 ± 0.32, a specificity of 0.95 ± 0.08, a PPV of 0.79 ± 0.32, an NPV of 0.86 ± 0.10, and a balanced accuracy of 0.77 ± 0.17, respectively.

In the radiogenomics-NB *ATRX*, female-specific model achieves an accuracy of 0.80 ± 0.11, a sensitivity of 0.79 ± 0.20, a specificity of 0.81 ± 0.15, a PPV of 0.76 ± 0.16, an NPV of 0.87 ± 0.12, and a balanced accuracy of 0.80 ± 0.12, respectively. The male specific *ATRX* model achieves an accuracy of 0.76 ± 0.12, a sensitivity of 0.69 ± 0.23, a specificity of 0.81 ± 0.14, a PPV of 0.73 ± 0.18, an NPV of 0.81 ± 0.12, and a balanced accuracy of 0.75 ± 0.13, respectively.

## Discussion

In this study, we propose a novel radiogenomics-NB model to fuse radiomics (imaging features) with RNAseq (genes) for glioma grading and prediction. NB distribution is appropriate for modeling RNAseq discrete read counts data and for preserving the count-based nature of this data. In the proposed radiogenomics-NB model, log-linear regression modeling is fitted to the estimated mean of the NB distribution and is linked with radiomics. We introduce this step to fuse the continuous radiomics data with the RNAseq count-based data without the need to transform the RNAseq data into a normal distribution.

The NB, unlike a Poisson distribution, has two parameters; the mean (e.g., the expected value of the RNAseq read counts data) and dispersion (e.g., a parameter that helps in capturing the variability of the RNAseq read counts). If the dispersion of NB is zero, the model reduces to Poisson distribution. In Poisson distribution, the mean is equal to the variance, which makes it rather restrictive. However, variation is usually observed in the real data of RNAseq counts data that the Poisson distribution cannot handle properly. On the other hand, NB has an additional parameter called the “dispersion” that allows the NB distribution of RNAseq counts data to modify its variance without affecting the mean. Thus, NB serves as a practical approximation to model RNAseq count data with variability different from its mean.

The mean of the proposed radiogenomics-NB model is estimated as the size factor multiplied by the total number of reads per RNAseq. Moreover, we utilize EdgeR to estimate the dispersion of the proposed radiogenomics-NB assuming RNAseq variability is assessed using the weighted conditional log-likelihood model. In the weighted conditional model, RNAseq counts data is assumed to have a distinct and individual dispersion for each RNAseq in addition to a common dispersion. Such an assumption can be more reliable when estimating the dispersion of real data of RNAseq counts data.

The performance evaluation of the proposed work indicates that linking simple, clinically feasible radiomics (i.e., tumor volumetric features) to RNAseq improves the performance of *IDH* and *ATRX* mutations prediction. The radiomics features utilized in the proposed radiogenomics-NB model that are described in Table 1 mainly depend on volumetric features. Our analysis shows that these features are associated with particular glioma mutations. This outcome supports previous studies that show the association between volumetric features and glioma mutations (32–35). The efficacy of the proposed radiogenomics-NB model is further investigated using the top 10, 20, 30, 50, 100, and 150 DERs, respectively. Our analysis shows that the smaller the number of DERs (fewer than 30 DERs) utilized in radiogenomics-NB, the better is the radiogenomics-NB model performance. Our analyses indicate that using fewer than 30 DERs in our analysis offers the best performance (statically significant) in the radiogenomics-NB codeletion and ATRX prediction model. This suggests that using large numbers of DERs (more than 30) in the proposed radiogenomics-NB would over parametrize the dataset and create model fitting problems and thus degrade the performance.

Comparing our radiogenomics-NB model to NBLDA, RF-genomics, FR-radiogenomics, and VoomNSC, our model significantly outperforms NBLDA, RF-genomics, and VoomNSC for prediction of *IDH* and *ATRX* mutations. Our radiogenomics-NB model offers similar performance as NBLDA, RF-genomics, RF-radiogenomics, and VoomNSC models for prediction of *1p/19q codeletion*. Specifically, for prediction of *IDH* mutations, while the proposed radiogenomics-NB model achieves significantly better balanced-accuracy, F1 score, and PPV than RF-radiogenomics, our model achieves similar accuracy, sensitivity, and specificity. Such results indicate the power of fusing radiomics and genomics data to develop radiogenomics models for classification and prediction models. The findings in this work indicate that the radiomics volumetric features may be vital for the prediction of *IDH* and *ATRX* mutations along with the genomics.

Different studies have revealed that gender is a significant factor in identifying cancer survival, prognosis, and treatment response (39–41). Hence, improved glioma molecular mutation prediction may require the development of gender-specific models. In this study, we explore the gender-specific effect on the radiogenomics-NB models. Our analysis reveals that *IDH* mutated patients remain significant after stratifying for gender, unlike *1p/19q codeletion* and *ATRX* status. Moreover, our analysis indicates that no association is found between gender and the three specific mutations (*IDH* mutations, *1p/19q codeletion*, and *ATRX* status) using the Chi-square test. This result is in agreement with the findings in Brat et al. (42), Li et al. (43), and Ebrahimi et al. (44). However, our gender-specific modeling shows that female-specific radiogenomics-NB models significantly outperform the male-specific radiogenomics-NB models for prediction of *IDH* status, *1p/19q codeletion*, and *ATRX* status, respectively.

In conclusion, we present a glioma mutations radiogenomics-NB prediction model that preserves the count nature of RNAseq counts data in the NB model and utilizes radiomics to develop a complete and a better characterization prediction model of patient data. Our analysis shows the superiority of utilizing both genomics and clinically feasible radiomics data when compared to only genomics models. Use of tumor volumetrics can be more easily and reproducibly implemented in clinical practice compared to more complex radiomics metrics, such as higher order texture analysis features. Finally, this study shows the efficacy of volumetric radiomics features in the radiogenomics-NB model for glioma molecular characterization and prediction. This study is a first step toward implementing joint modeling of RNAseq and MRI patient data for glioma grading. However, further investigation is needed with a larger dataset with both RNAseq and full multimodality MRI dataset for each patient in a cohort. In the future, larger prospective studies may be needed to investigate specific radiomics features and their association with the different mutations and RNAseq read counts data for implementation into clinical workflow. Furthermore, it will be interesting to investigate the cause of superior performance of female-specific radiogenomics-NB models when compared to that of the male-specific radiogenomics-NB models for prediction of *IDH* status, *1p/19q codeletion*, and *ATRX* status. Also, these models may be further investigated in treatment response and survival prediction in the future.

## Data Availability Statement

Radiomics data are available at doi: 10.7937/K9/TCIA.2017.GJQ7R0EF. Genomics and mutations data are available at: https://portal.gdc.cancer.gov/.

## Ethics Statement

The studies involving human participants were reviewed and approved by Old Dominion University IRB. The ethics committee waived the requirement of written informed consent for participation.

## Author Contributions

ZS and KI: conception and design and development of methodology. ZS, ND, and KI: analysis and interpretation of data. ZS, ND, AV, JC, and KI: drafting the article and/or revising. KI: funding acquisition. All authors contributed to the article and approved the submitted version.

## Conflict of Interest

The authors declare that the research was conducted in the absence of any commercial or financial relationships that could be construed as a potential conflict of interest.

## Publisher's Note

All claims expressed in this article are solely those of the authors and do not necessarily represent those of their affiliated organizations, or those of the publisher, the editors and the reviewers. Any product that may be evaluated in this article, or claim that may be made by its manufacturer, is not guaranteed or endorsed by the publisher.
